# The Counter Effect of Exercise on Cisplatin-Induced Cognitive and Proliferation Impairments

**DOI:** 10.7759/cureus.52526

**Published:** 2024-01-18

**Authors:** Maha Elbeltagy, Ramzi A Al-horani, Tala S Alsharaeh, Amro H Alkhatib, Ibrahim Alawaisheh, Ahmad A Abuhani, Ahmed Salman

**Affiliations:** 1 Department of Anatomy and Histology, Faculty of Medicine, The University of Jordan, Amman, JOR; 2 Department of Anatomy, Faculty of Medicine, Menoufia University, Shibin El Kom, EGY; 3 Department of Exercise Science, Yarmouk University, Irbid, JOR; 4 Department of General Medicine, University of Jordan Hospital, Amman, JOR

**Keywords:** ki-67, chemobrain, cognition, neurogenesis, cisplatin

## Abstract

Background

Cisplatin, a widely used chemotherapeutic agent, offers therapeutic benefits for cancer treatment but often leads to adverse effects on neurogenesis and oxidative stress, causing cognitive impairment. Concurrent physical activity has been proposed as a potential strategy to counteract these side effects. This study aimed to investigate the impact of physical exercise on cisplatin-induced cognitive impairment in a mouse model.

Methods

Adult male mice (n=45) were divided into three groups: control, cisplatin-treated (2.3 mg/kg), and exercise/cisplatin. Cisplatin was administered intraperitoneally over one month, while the exercise/cisplatin group underwent moderate-intensity exercise alongside cisplatin treatment. Spatial memory was evaluated using the novel object recognition (NOR) task, and hippocampal proliferation and oxidative stress were examined using Ki-67 and glutathione peroxidase (GPx) immunohistochemistry (IHC) staining, respectively. Statistical analyses were performed using the GraphPad Prism 4.0 software (GraphPad Software, San Diego, CA).

Results

The cisplatin-treated mice exhibited significantly lower preference index (PI) scores in the NOR task compared to the control (p<0.001) and exercise/cisplatin (p<0.001) groups. IHC staining revealed impaired hippocampal proliferation and increased oxidative stress in the cisplatin-treated group relative to the control and exercise/cisplatin groups. The introduction of a moderate-intensity exercise protocol appeared to mitigate the decline in hippocampal proliferation and oxidative damage induced by cisplatin. Additionally, cisplatin-treated mice experienced weight loss, while exercise attenuated this effect.

Conclusion

Cisplatin treatment resulted in decreased memory, hippocampal proliferation, and weight loss in mice. Concurrent moderate-intensity exercise seemed to alleviate these effects, suggesting a potential role for physical activity in ameliorating cisplatin-induced cognitive decline. This study underscores the importance of incorporating exercise as a complementary strategy to enhance cognitive outcomes in cancer patients undergoing cisplatin treatment.

## Introduction

Cancer is defined as the uncontrolled growth of abnormal cells in any part of the body [1]. It was the second leading cause of death in the United States in 2019, and its global impact continues to grow, putting physical, emotional, and financial burdens on all parties involved [2]. Surgical procedures, radiation, and drugs are all standard commonly used cancer treatments.

Chemotherapeutics are one example of drugs used to prevent cancer cells from dividing uncontrollably. One of the commonly used platinum-based chemotherapeutic drugs is cisplatin. Cisplatin is a well-known chemotherapeutic agent that has been used as a standard treatment for many malignancies including bladder, head and neck, and non-small cell lung cancer [3]. Despite cisplatin's therapeutic benefits, toxicities are frequently reported side effects after treatment [4]. Cancer patients treated with cisplatin often experience cognitive impairments [5-7]. The underlying, possible mechanisms of cisplatin-induced side effects are still being explored, although there are several possible etiologies. According to research, cisplatin appeared to decrease the expression of brain-derived neurotrophic factor (BDNF), as well as spine density, dendritic branching, and neurogenesis in the hippocampus of rats [8,9]. Furthermore, when adult rats were given cisplatin, hippocampal neuronal tissue showed increased mitochondrial DNA damage, oxidative damage, and activated caspase-9 [9].

Physical exercise has long been proven to be beneficial to one's health in a variety of ways [10]. Exercising can reduce oxidative stress according to several new studies through the regulation of catalase and glutathione activity, superoxide dismutase (SOD) activity, and reactive oxygen species (ROS) [11]. In other words, exercise training appears to have an antioxidant effect, which has been shown to help with a variety of clinical issues [12-14]. Based on the aforementioned evidence, exercise has been used in several trials to reverse the side effects of radiation and chemotherapy. Aerobic exercise has been shown to minimize cancer-related decline resulting in a significant improvement in cancer survivors' quality of life [15]. The ameliorating effects of physical exercise on doxorubicin-induced cognitive impairment have been demonstrated in previous experimental studies [16].

Having safe and affordable methods to counteract the harms induced by cisplatin would be of great benefit in improving the quality of life of patients receiving cisplatin therapy. One of the proposed methods is through concurrent physical exercise, which was proven to stimulate neurogenesis and reduce oxidative stress [11,17]. Consequently, this study aimed to establish a mouse model of cisplatin-induced cognitive impairment and investigate how moderate-intensity physical activity affected cisplatin-induced cognitive dysfunction and whether it alleviates cisplatin-induced cognitive decline or not.

## Materials and methods

Animals, drug preparations, and exercise protocol

After two weeks of habituation in the vivarium, 45 male mice (six weeks old, 20-30 g) were purchased from Yarmouk University Animal Center (Amman, Jordan). All animal experiments were approved by the Scientific Research Ethics Committee, Deanship of Scientific Research, The University of Jordan (number: 308/2021/19).

The mice were randomly assigned to three groups: control (n=15), cisplatin-treated (n=15), and exercise/cisplatin groups (n=15). The food consumption was determined daily by weighing the additional food using an electronic balance. A portion of 5 g per mouse per day was added. The following day, the remaining food was weighed, and the difference from the added quantity was calculated to establish the daily food intake. Regular observations were conducted to identify signs of illness and injuries resulting from conflicts. Aggressive mice were segregated into separate cages, and any wounds were promptly treated with iodine on a daily basis for a week excluding injured mice from the ongoing study. The weekly weight measurements of the mice were consistently taken at the same time. All mice were housed in groups of two to three with free access to food and water under a 12-hour light to 12-hour dark cycle.

The cisplatin-treated mice received 15 intraperitoneal injections (2.3 mg/kg/week) over four weeks, with mannitol administered to mitigate nephrotoxic effects. This treatment and the corresponding dosage were chosen based on previous literature [9]. The control mice received an identical saline dosage.

A moderate-intensity exercise program was adopted from previous literature [18] with some modifications, and a rodent treadmill was utilized. To assess the exercise capabilities of mice, the maximum distance covered during exhaustive exercise was recorded in compliance with previous studies [18]. The mice in the cisplatin/exercise group underwent acclimatizing treadmill exercise sessions, occurring four times a week for 10-25 minutes each day, with a slope of zero and a speed ranging from 15 to 30 cm/second. Following adaptation sessions, the mice underwent exercise training for four weeks. The moderate-intensity treadmill exercise was carried out four days a week and consisted of 35-50 minutes a day in the first two weeks and 50-60 in the last two weeks, with a slope of zero. The speed in the first week was started at 10 cm/second and was increased gradually over the subsequent weeks to 35 cm/second. Noncompliant mice were excluded. The control and cisplatin-treated groups spent equivalent time on nonoperational treadmills to mitigate the influence of additional environmental variables on the outcomes.

Novel object recognition (NOR) test

**Figure 1:**
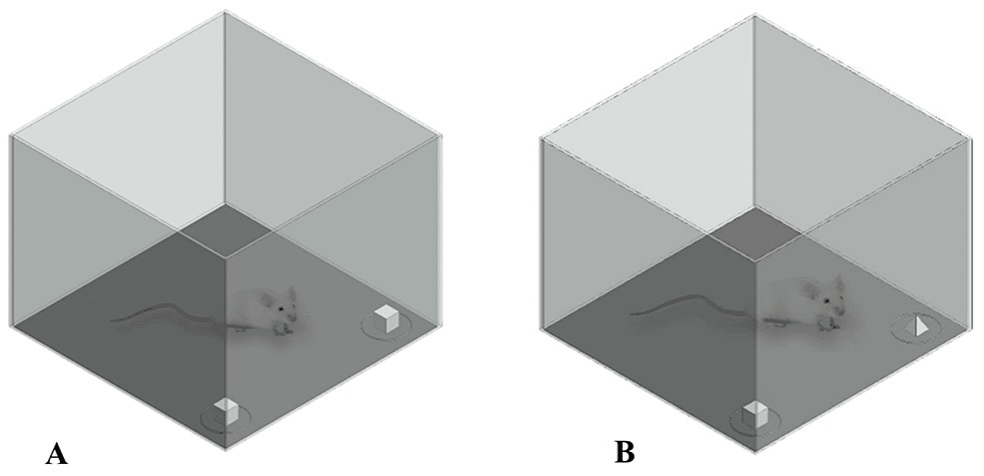
NOR test experimental setup. Panel A: Familiarization phase where two identical objects are placed for a mouse to recognize. Panel B: The testing phase where one object will be replaced with a novel object. NOR: novel object recognition

Cognitive function in mice was assessed using the novel object recognition (NOR) test before and after the last injection. Following Ennaceur and Delacour's method (1988), the test used plastic boxes (85×85×65 cm) where mice were habituated for one hour [19]. During the familiarization trial, two identical objects were placed, and the animals explored for three minutes. After a five-minute interval, one object was replaced with another object of similar dimensions (novel object), while the other was left unchanged (familiar object) in the choice trial. Object exploration (sniffing, licking, and chewing) at a distance of ≤1 cm was recorded [20]. The total time on each object was measured, and a discrimination index was calculated as the difference in time spent on familiar and novel objects divided by the total exploration time (Figure 1) [21].

Euthanization, brain tissue preparation, and immunohistochemistry (IHC)

Antioxidative effects and hippocampal cell survival were assessed via Ki-67 and glutathione peroxidase (GPx) immunohistochemistry. Mice, euthanized with ether inhalation after behavioral tests, had their brains removed, trimmed, and fixed using a 3% glutaraldehyde solution overnight and were then embedded in paraffin as 4 µm-thick sections using a Leica microtome (Leica RM2235 Microtome, Leica Biosystems, Wetzlar, Germany). These sections were placed on positively charged slides for standard staining with hematoxylin and eosin, as well as for the immunohistochemical analysis of Ki-67 and GPx.

For Ki-67 immunostaining, sections underwent epitope retrieval in a 95°C water bath with Tris-ethylenediaminetetraacetic acid (EDTA) solution (0.01 M and pH 9.0) for an hour, while GPx1 sections were retrieved in a 95°C water bath with sodium citrate solution (0.01 mM and pH 6.0) for 25 minutes. After retrieval, sections were rinsed, treated with 3% hydrogen peroxide at room temperature for 10 minutes, and washed with phosphate buffer solution (PBS, 0.1 M and pH 7.4). To prevent nonspecific binding, sections were blocked with 5% bovine serum albumin in PBS for an hour, followed by overnight incubation at 4°C with GPx1 antibody (rabbit polyclonal, 1:750 dilution, Invitrogen, Waltham, MA) and Ki-67 antibody (mouse monoclonal, 1:100 dilution, Abcam, Cambridge, United Kingdom), diluted in PBS with 0.2% Tween 20 detergent.

After primary antibody incubation, sections were washed with PBS, incubated with a complement reagent (ab236466, Abcam, Cambridge, United Kingdom) for 10 minutes, followed by rinsing and incubating with goat anti-rabbit linker (ab236466, Abcam, Cambridge, United Kingdom) for 15 minutes. Color development was achieved with a six-minute diaminobenzidine (DAB) treatment, followed by a five-minute hematoxylin counterstaining. Slides were dehydrated with increasing ethanol concentrations, cleared with xylene, and cover-slipped using dibutyl phthalate polystyrene xylene (DPX) mounting media.

For quality control, positive and negative control slides were included in each staining run, utilizing mouse intestinal epithelium tissue sections as positive controls for Ki-67 and GPx1. Negative controls were prepared by excluding the primary antibody and using PBS as a substitute. Positive staining for GPx1 displayed a dark blue-purple cytoplasmic pattern, while positive staining for Ki-67 exhibited a dark brown nuclear pattern.

Statistical analysis

GraphPad Prism 4.0 software (GraphPad Software, San Diego, CA) was used to calculate all statistical parameters. The NOR task data was analyzed using a paired Student's t-test (two-tailed) and repeated-measures analysis of variance (ANOVA), while the proliferating cell count and animal preference index (PI) data were analyzed using one-way ANOVA with Bonferroni's post hoc test. Statistical significance was defined as a p-value of <0.05.

## Results

The effect of cisplatin on the novel object recognition task

**Figure 2:**
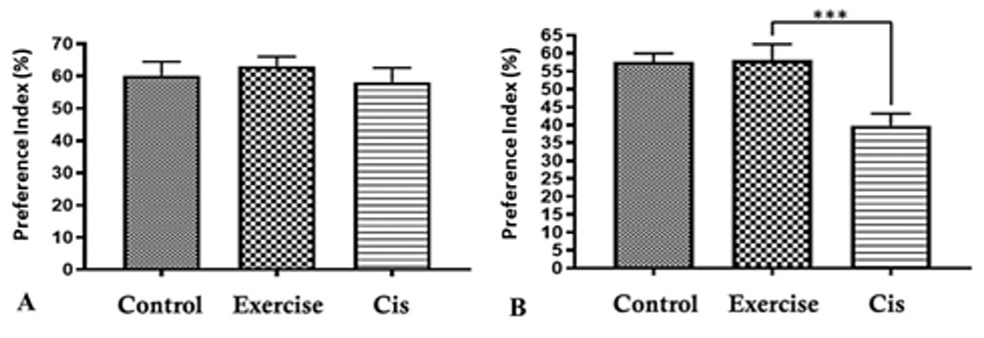
The preference index (PI) is defined as the calculated exploration time of the new location, represented as a percentage of the combined exploration time of both novel and familiar locations of an object. The analysis used was one-way ANOVA with Bonferroni's post hoc test to assess the difference. Panel A: The PI index among the groups at baseline showing comparable results. Panel B: The PI index after treatments showing that mice treated with cisplatin (Cis) alone had a decline in the PI, which appeared to be significantly improved by exercise (p<0.01). ***Statistically significant difference. ANOVA: analysis of variance

In the familiarization trial, both the control and treatment groups exhibited equal exploration times for each object. In the choice trial, the control and exercise/cisplatin groups significantly favored the novel object over the familiar one. However, the cisplatin-treated group did not display a significant difference in exploration times between the objects (Table 1). A significant decline in the preference index (PI) was observed in the cisplatin-treated group compared to the exercise/cisplatin group (p<0.01) (Figure 2).

**Table 1:** Preference index in all mice groups at baseline and after the experiment. The preference index was calculated as the difference between the time spent exploring the novel object and the time spent exploring the familiar object divided by total exploration time and presented as a percentage.

Mouse Number	Control		Cisplatin Only		Cisplatin/Exercise	
	Baseline (%)	Post-treatment (%)	Baseline (%)	Post-treatment (%)	Baseline (%)	Post-treatment (%)
1	68	57.6	44	32	56.5	51
2	78	53	63	29	75.5	63
3	68	68	71	56	62	71
4	64	56	88	40	72	-
5	53	59	73	45	63	-
6	59	50	65	35	66	88
7	79	76	44	49	74	73
8	59	57	43	34	57	64.5
9	57	57	51	35	35	47
10	47.5	45.5	49	29	58	43
11	29	59.5	34	45	65	51
12	61	47.6	43	41	63	49
13	64	63	60	39	73	45
14	60	57	92	36	64	46
15	-	-	51	41	62	65

Effect of different treatments on the weights of animals

**Figure 3:**
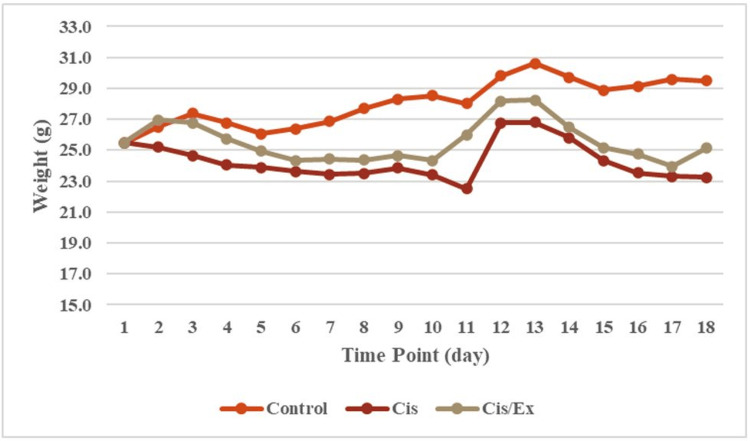
The difference in animal body weight in grams across groups during the injection period. On a GraphPad Prism 4, the analysis was conducted using a two-way ANOVA with Bonferroni's post hoc test to compare the means showing a significant difference between the groups (p<0.001). ANOVA, analysis of variance; Cis, cisplatin; Ex, exercise

The food intake and body weight of mice in both groups were measured over four weeks (Appendices) and analyzed using repeated-measures ANOVA. The treatment resulted in a noteworthy intergroup difference (p<0.001) (Figure 3). Mice subjected to cisplatin treatment exhibited apparent weight loss. Over the injection duration, the weight of cisplatin-treated mice was significantly lower than that of their saline-treated counterparts (p<0.001). Physical activity seemed to significantly mitigate the observed weight loss (p<0.001).

Effect of treatments on proliferating cell counts and antioxidant activity

**Figure 4:**
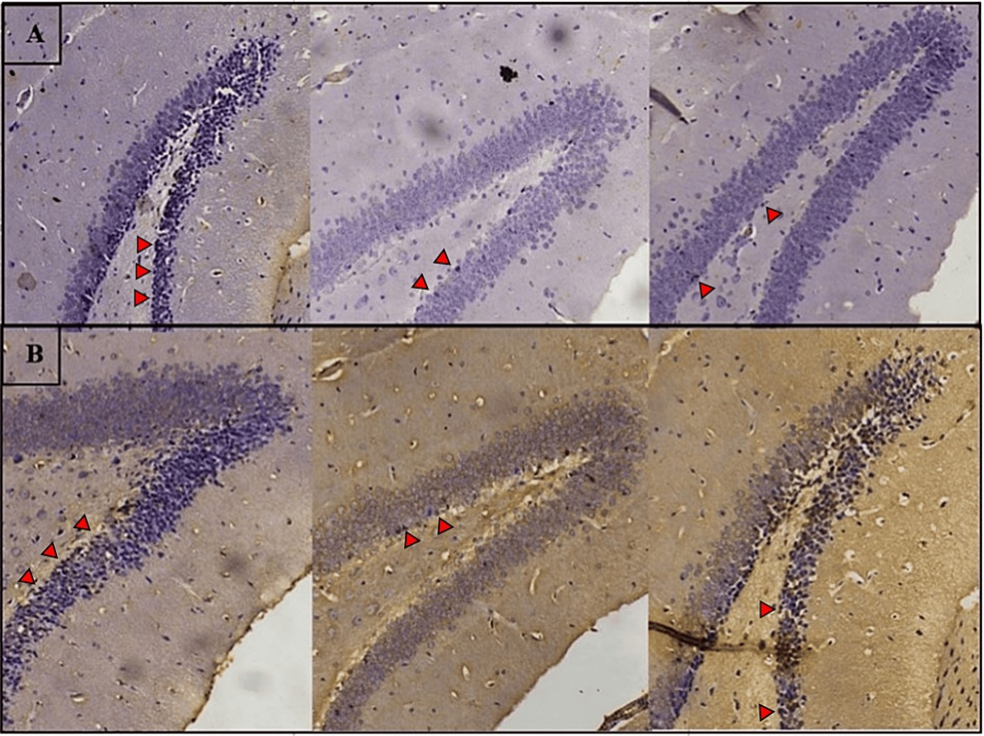
Immunohistochemistry staining of the dentate gyrus cells of examined mice from the control (left), cisplatin (middle), and exercise/cisplatin groups (right). Panel A: Proliferating cells (arrows) within the dentate gyrus immunostained for Ki-67. Panel B: GPx antioxidant activity in cells (arrows). GPx: glutathione peroxidase

Figure 4A displays a representative image of Ki-67-positive proliferating cells in the dentate gyrus, counterstained with propidium iodide. The cisplatin-treated group exhibited a lower total number of Ki-67-positive cells (cell count range: 87-95) compared to the control group (cell count range: 113-126). This reduction was mitigated by simultaneous exercise co-treatment (cell count range: 101-119). In Figure 4B, GPx-positive cells, indicating oxidative stress levels in the dentate gyrus, are depicted. The cisplatin-treated group demonstrated elevated oxidative stress levels (cell count range: 11-15) compared to the control group (cell count range: 20-26). Co-treatment with exercise appeared to significantly enhance antioxidant activity (cell count range: 23-30).

**Figure 5:**
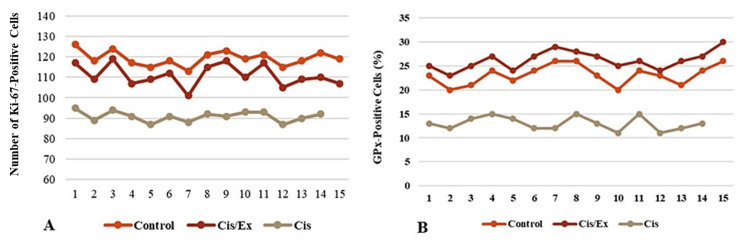
A) Average number of Ki-67-positive cells in the dentate gyrus of the included mice in each group. The higher the number of Ki-67-positive cells, the more cells are actively dividing. A significant difference was found in the mean number of Ki-67-positive cells between all groups using one-way ANOVA test (p<0.01), revealing a significant ameliorating effect of exercise (Ex) on the cell count reduction caused by cisplatin (Cis) (p<0.01). B) GPx activity percentage in the dentate gyrus of mice in each group. GPx activity appeared to be significantly higher in the Cis/Ex group compared to the other groups (p<0.001). ANOVA, analysis of variance; GPx, glutathione peroxidase

A substantial reduction in the total number of Ki-67-positive cells in the cisplatin-treated group was observed compared to the control group (p<0.01), which was ameliorated by co-treatment with the exercise protocol (p<0.01) (Figure 5A). GPx activity was significantly higher in cisplatin-treated mice group who underwent exercise compared to those in the control or cisplatin groups (p<0.001) (Figure 5B).

## Discussion

This experimental study revealed that administering cisplatin led to significantly diminished memory, hippocampal proliferation, and weight loss in mice. Chemobrain describes cognitive and memory difficulties that a cancer patient may experience due to therapy [22]. It affects the recall of both semantic and episodic parts of memory, both of which are known to require hippocampal development [23]. One study calculated the frequency of cognitive decline among 22 testicular cancer patients treated with cisplatin. Cognitive impairment was estimated to be 63.6% of the cisplatin-treated group, with verbal learning and memory being the most affected (13.6% and 28.6%, respectively) [24]. The underlying, possible mechanisms of cisplatin-induced side effects are still being explored, although there are several possible etiologies. For instance, according to Lomeli et al. [9], cisplatin seemed to decrease serum BDNF levels, as well as spine density, dendritic branching, and neurogenesis in the hippocampus of rats [8]. There are other findings that further support the fact that cisplatin induces neuronal apoptosis, that is, decreased neurogenesis [25]. Marullo et al. observed that, eventually, cisplatin induces cytochrome c release, which contributes to apoptosis [26].

In this study, the NOR test was applied to explore hippocampal function to detect the cognitive consequences of exercise in cisplatin-treated mice [19]. The effect of cisplatin on cognition was demonstrated in this study, showing that mice who were injected with cisplatin showed a decreased PI compared to baseline and indicating that cisplatin treatment caused their memory and cognition to decline significantly. Moreover, a significant reduction in the PI was noticed among the cisplatin-treated group compared to the exercise/cisplatin group. These findings indicate that the mice subjected to an exercise protocol along with cisplatin injections had better cognition compared to those treated with cisplatin only.

Exercise appears to have controversial effects on oxidative stress. A systematic review concluded that 16 studies showed a decrease in oxidative stress while only seven reports revealed an increase in oxidative stress [11]. One study stated that high-intensity exercise increases oxidative damage in contrast to moderate intensity [27], which is why we utilized a moderate-intensity exercise protocol and used glutathione peroxidase activity as an indicator of oxidative stress. The tissue samples obtained from the cisplatin group demonstrated decreased activity of GPx compared to mice who underwent the exercise protocol. Thus, it appears that exercise alleviated oxidative damage caused by cisplatin.

It has been shown that exercise improves neurogenesis. Some studies investigated the change in BDNF and tropomyosin receptor kinase B (TrkB) levels in rodents and concluded that exercise increases their expression, further contributing to the enhancement of neuroplasticity and neurogenesis especially in the hippocampus [28-31]. Testing the changes in the levels of hippocampal proliferation markers is a supposedly suitable way of testing the effects of different variables on cognitive impairment, namely, memory and its many subtypes. Ki-67 is a direct proliferation marker found to be markedly reduced in the hippocampal samples of cisplatin-treated animals [32] and, on the other hand, increased in those given an exercise regimen [33]. In this study, we were able to support both findings concurrently where the total number of positive Ki-67 cells was higher among the exercise group in comparison to the cisplatin group, supporting the effect of exercise in protecting the hippocampal neurogenesis from cisplatin's toxic effects.

While our study provides valuable insights into the benefits of exercise in alleviating cisplatin-induced cognitive impairment, it is crucial to acknowledge several limitations that may influence the interpretation and generalizability of our findings. Firstly, the use of mice as experimental subjects introduces the possibility of species-specific differences, and it is difficult to extrapolate these results to humans. Because humans and animals exhibit different indicators of toxicity, performing research on animals may limit the applicability of study findings to humans [34]. Secondly, the controlled laboratory environment may not fully replicate the dynamic conditions found in real-world settings, and the small sample size in our study could impact the statistical power and reliability of the results [35]. Furthermore, the age of the mice used in the experiment may not represent the entire lifespan or developmental stages of the species. While our findings contribute to the current understanding of exercise advantages, further research addressing these limitations is necessary to enhance the robustness and applicability of our conclusions.

## Conclusions

In conclusion, this study showed that cisplatin treatments caused cognitive deficits and decreased hippocampal neurogenesis in the dentate gyrus, and these effects were ameliorated when cisplatin-treated mice underwent a moderate-intensity protocol, causing a reduction in oxidative damage and apoptosis. The results of this study may serve as a springboard for further research into practical approaches to reduce chemotherapy-induced deterioration.

## Appendices

Table 2 shows the body weight changes of mice measured over four weeks.

**Table 2:** Mice weight changes in grams over the four-week duration of the experiment.

Control Group																																
Mouse Number		Initial Weight			Week 1									Week 2							Week 3									Week 4		
					Day 1	Day 2		Day 3		Day 4		Day 5		Day 1	Day 2		Day 3		Day 4		Day 1	Day 2		Day 3		Day 4		Day 5		Day 1	Day 2	
1		24.3			29.3	29.3		29.3		29.7		29.7		29.7	29.9		30		30.1		30.8	31.1		30.5		29.8		30.1		30.4	31.5	
2		21.2			27.6	26.3		26.7		27.6		27.6		28	28.7		29.8		29.8		31.8	32.1		31		29.6		30.7		30.8	30.7	
3		22.7			28.1	27.1		22.3		25.6		25.6		26	28		28		28		28.2	28.6		28.1		27.1		27.7		27.4	28.3	
4		29.59			29.7	27.6		27.4		27.3		25.6		29	30		30		30.4		30.6	31.2		30.5		30.1		30.3		30.6	30.5	
5		23.5			25.3	25.2		23.6		23.6		27.9		25.3	26		26.3		26.4		27.5	31		27.3		26.8		26.8		25.4	26.2	
6		22.47			23.1	23.2		23.2		23.6		25		24.3	25.6		25.8		26		26.5	27		26.1		25.5		25.2		30.2	25.5	
7		29.1			24.3	24.3		23.4		23.3		24		31	31		30.1		32.1		34.3	33.6		33.5		32.8		33.2		33.2	33.4	
8		30.2			26.9	26.3		24.2		24.3		23.4		26.1	26.2		28.6		28.9		30.3	30.1		30.5		28.5		30		30.2	30.1	
9		26.2			26.6	26.4		25.7		25.3		25.3		26.2	27.3		27.8		27.9		29.4	30.8		28.9		29		26.7		28.3	28.5	
10		22.3			29.6	29.4		29.4		30.1		31.1		31.1	30		30		30		30.8	32.2		31.6		30.4		30.5		31.3	31.2	
11		29.8			25.3	24.9		25.3		25.9		25.9		26.1	28		25.9		25.9		27.1	27.3		27.8		26.6		26.9		27.2	27.4	
12		20.19			25.6	25.3		25.5		24.5		24.5		24.3	24.4		24.6		25.9		24.2	25.3		24.5		24.1		24.5		24.5	24.3	
13		29.1			34.3	32.1		31.4		31.4		32.1		32.1	32.2		32.5		32.5		35.6	37.2		35.7		35.1		35.3		35.1	35.3	
14		25.6			27.4	27.2		27.4		27.3		28.2		28.7	29.1		30		29.8		30.2	31		30.1		28.8		29.9		29.6	29.9	
Cisplatin-Only Group																																
Mouse Number	Initial Weight				Week 1									Week 2							Week 3									Week 4		
					Day 1		Day 2		Day 3		Day 4		Day 5	Day 1		Day 2		Day 3		Day 4	Day 1		Day 2		Day 3		Day 4		Day 5	Day 1		Day 2
1				20.24	21.3		20.1		20		20		20	20		20.7		20.6		20.4	23.6		23.6		22		20.1		19.6	18.5		20
2				28.7	26.7		23.6		22.3		21.9		21.4	21.4		21.5		21.3		23.1	27.4		27.1		26.7		24.8		25.6	23.9		24.7
3				24.8	29.2		23.4		23.3		22.9		22.9	22.9		23.1		23.1		24.2	29.8		30.6		28.9		27.9		27.1	25.5		25.8
4				28.7	29.1		29.1		28.4		26.2		25.7	24.1		24.3		24.2		24.5	31.3		31		30		28.1		27.3	25.7		27.9
5				29.4	28.1		28.2		26.4		28.9		28.9	28.7		28.7		28.7		27.6	37.0		36.9		34.8		33.5		32.5	31.4		32.8
6				25.9	27.6		26.7		26.2		26.4		26.4	25.9		25.8		25.8		25.8	27.3		27.1		25.4		24.7		23.3	21.5		24
7				24.3	31.2		31.2		28.9		27.2		29.1	30		30		28.1		28.4	30.6		31.3		29.7		28.5		28.2	27.5		29.8
8				25.7	29.6		29.2		26.7		26.7		26.7	26		26.1		26		26	29.3		29.6		26.7		25.6		25.8	27.6		28.2
9				26.87	23.6		23.1		22.8		22.1		22.4	23		25.6		25.6		25.7	30.3		29.3		25.4		24.7		25.4	27.3		28.2
10				22.03	27.6		27.4		22.8		22.4		22.2	22.4		22.5		23.7		23.5	28.6		28.5		26.5		25.3		24.8	24.5		25
11				26.5	26.7		26.1		27.4		22.6		24.3	24.3		24.8		24.1		22.7	25.3		25.3		23.7		22.6		24	21.2		23.3
12				25.02	25.6		25.4		26.5		26.2		25.4	25.4		25.7		24.2		24.2	27.3		26.5		25.6		24.5		22.5	23.4		23.5
13				26.4	27.3		27.2		27.3		26.2		26	26.3		25.2		25.2		25.2	28.7		29.3		27.5		26		25.5	23.8		24.7
14				24.6	24.5		24.3		22.6		22.8		22.2	22.7		23.4		22		22.1	22.4		23.1		21.6		19.9		19.3	17.7		18.5
15				23.2	23.4		21.3		22.3		22.5		22.6	22.4		22.4		22.2		22.3	23.8		24.3		22.6		21.2		20.4	19.6		20.5
Cisplatin/Exercise Group																																
Mouse Number			Initial Weight		Week 1									Week 2							Week 3									Week 4		
					Day 1		Day 2		Day 3		Day 4		Day 5	Day 1		Day 2		Day 3		Day 4	Day 1		Day 2		Day 3		Day 4		Day 5	Day 1		Day 2
1			20.4		25.7		24.8		22.7		22.7		22.6	22.3		22.4		22.2		22.1	26.3		26.1		25.5		23.6		23.9	24.6		25.7
2			26.4		23.7		23.2		24.6		23.2		21.4	23.3		23.4		23.4		23.2	26.8		25.9		25.1		23.4		23.2	22.1		23.5
3			23.4		20.6		20.3		21.4		20.2		22.9	20.3		20.1		19		19	19.5		21.1		20.2		19.8		18.8	18.9		18.9
4			23.04		-		-		-		-		-	-		-		-		-	-		-		-		-		-	-		-
5			22.3		-		-		-		-		-	-		-		-		-	-		-		-		-		-	-		-
6			26.7		27.7		27.6		27.7		27.2		26.1	26		26		24.9		24.9	27.5		26.4		25.6		23.8		23.4	25.3		25.7
7			24.7		23.4		22.3		22.3		22.2		22.2	21.1		21.9		21.7		22.7	24.3		23.4		23.6		21.3		21.5	20.2		19.9
8			23.6		24.7		23.5		22		22.3		22.2	22.1		22.9		22.8		22.5	25.9		25.5		24.7		23.2		22.3	22.1		21.5
9			24.6		26.5		26.2		26.5		26.2		26.2	25.4		25.6		25.4		24.3	26.5		26.9		26.3		24.5		23.8	22.3		22
10			30		26		25.6		25.6		26.3		26.1	26		26.5		24.9		24.2	30.7		29.4		28.3		27.3		24.7	25.1		25.7
11			26.7		22.3		22.3		21.3		21.3		20.9	20.9		22.3		22.4		22.4	25.3		24.9		23.6		21.8		20.4	21		20.5
12			27.12		27.6		27.4		26.5		25.9		24.7	24.7		24.7		24.6		24.5	30.0		29.3		28.7		27.2		25.7	24.9		25.2
13			26.6		22.9		22.1		22.5		22.5		22.5	25.2		25.3		26.1		26	26.7		28.3		27.2		25.9		26.5	24.3		23.9
14			25.4		26.2		24.3		24.3		24.2		24.2	25.1		25.9		24.8		24.7	29.5		30.4		28.5		27.1		25.5	25.1		24.9
15			28.9		23.1		23.1		22.9		22.9		22.6	23.1		23.2		22.2		23.3	29.1		30.7		28.2		27.3		26.1	26.9		24.7
